# Non-Surgical Strategies for Assisting Closure of Pharyngocutaneous Fistula after Total Laryngectomy: A Systematic Review of the Literature

**DOI:** 10.3390/jcm11010100

**Published:** 2021-12-24

**Authors:** Luca Giovanni Locatello, Giuseppe Licci, Giandomenico Maggiore, Oreste Gallo

**Affiliations:** 1 Department of Otorhinolaryngology, Careggi University Hospital, Largo Brambilla, 3, 50134 Florence, Italy; giuseppe.licci@unifi.it (G.L.); maggiore2@virgilio.it (G.M.); oreste.gallo@unifi.it (O.G.); 2 Department of Experimental and Clinical Medicine, University of Florence, 50134 Florence, Italy

**Keywords:** head and neck, fistula, complications, non-surgical treatment, otorhinolaryngology

## Abstract

Background: Pharyngocutaneous fistula (PCF) is a frequent complication after total laryngectomy, with an incidence of up to 65%. Many conservative or invasive approaches are available and the choice among them is usually made on a case-by-case basis. The aim of the present review is to critically summarize the available evidence of the effectiveness of the non-surgical management of PCF. Methods: A systematic review and a meta-analysis of the literature were conducted, according to the PRISMA guidelines. Studies investigating botulinum toxin therapy, scopolamine transdermal patch, hyperbaric oxygen therapy (HBOT), and negative pressure wound therapy (NPWT) were assessed. Complete fistula closure after the initiation of non-surgical treatment was the main outcome. Results: After the application of selection criteria, a total of seven articles and 27 patients were included in the present review. All the eligible studies were descriptive case series, while only one article used a standard group as a comparison. The mean age was 63.3 and 14 patients (51.9%) had previously received RT. The reported comorbidities were diabetes, ischemic heart disease, hypertension, dyslipidemia, COPD, and atrial fibrillation. With a mean healing time of 25.0 days, the overall success rate was 92.6%. Conclusions: Non-surgical treatment of PCF is only based on the experience of small series. Although success rates seem promising, the absence of properly designed comparative studies does not allow us, at present, to identify ideal candidates for these non-invasive management strategies for PCF.

## 1. Introduction

Pharyngocutaneous fistula (PCF) is a frequent complication after total laryngectomy, with a reported incidence between 3% and 65% [1]. This event considerably increases the length of hospital stay and costs, may delay the start of postoperative radiotherapy (RT), and can heavily affect the patient’s psychological status [2]. PCF is usually diagnosed 7 to 11 days after surgery [2], and while there is still no gold standard test (e.g., blue dye test, etc.) for an early diagnosis [3], fever in the early postoperative period represents an excellent predictor of its development [4]. 

Once PCF is diagnosed, standard wound treatment is usually implemented, in terms of compressive dressings, antibiotics coverage, and artificial nutrition. Closure can be expedited by invasive/surgical approaches (use of pedicled or free flaps, endoscopic repair) or by non-surgical strategies, such as the use of hyperbaric oxygen therapy, botulinum toxin injection, or negative pressure (or vacuum-assisted) therapy [1,5]. While excellent reviews have been published on the management of post-reconstructive head and neck salivary fistulas [5], or about the reduction strategies of salivary flow in head and neck cancer patients [6], no specific paper has ever focused on non-surgical treatment of PCF, which remains largely empirical and anecdotal. In the present review, we want to critically summarize the available evidence on the efficacy of the non-surgical treatments of PCF after total laryngectomy.

## 2. Materials and Methods

### 2.1. Literature Search

Following the preferred reporting items for systematic reviews and meta-analyses (PRISMA) guidelines [7], we conducted a literature search on articles published from January 1980 up to July 2021, using the PubMed database in order to identify all the studies reporting the outcomes of non-surgical strategies for the treatment of PCF. 

The following keywords were used: “treatment AND pharyngocutaneous fistula” (596 results, 517 in English, 1 in Italian); “Management AND pharyngocutaneous fistula” (185 results, 167 in English, 0 in Italian); “Closing AND pharyngocutaneous fistula” (78 results, 67 in English, 1 in Italian); “Botulinum AND pharyngocutaneous fistula” (2 articles, 2 in English); “Botulinum AND salivary fistula” (47 articles, 40 in English, 0 in Italian); “Botulinum AND saliva” (172 articles, 149 in English, 0 in Italian); “Hyperbaric oxygen therapy AND pharyngocutaneous” (4 articles, 3 in English, 0 in Italian); “Oxygen AND pharyngocutaneous fistula” (4 articles, 3 in English, 0 in Italian); “HBOT AND pharyngocutaneous fistula” (1 article, 1 in English); “Vacuum Assisted Closure Therapy AND pharyngocutaneous fistula” (11 results, 9 in English, 0 in Italian); “Negative pressure wound therapy AND pharyngocutaneous fistula” (16 results, 13 in English, 0 in Italian); “Scopolamine AND pharyngocutaneous fistula” (0 results); “Scopolamine AND fistula” (13 results, 13 in English, 0 in Italian).

Only studies describing the clinical outcomes of patients presenting with a PCF and who were exclusively treated with non-surgical methods were selected. Articles were excluded based on the following criteria: studies describing other types of fistulas or wounds in the head and neck region (e.g., oro-cutaneous fistulas, post-parotidectomy fistulas, etc.); cases with the concurrent implementation of an invasive approach (for example, vacuum-assisted closure therapy/VAC and the associated need for returning to the operating room, or the need of general anesthesia); and articles written in languages other than Italian and English. In order to avoid unnecessary bias, cases reporting combined medical treatments for a single-treatment-resistant fistula were also excluded. Bibliographic research and the removal of duplicates were performed by the reference management software Mendeley Version 1.19.8 for macOS. 

### 2.2. Data Collection

Titles and abstracts of the extracted papers were carefully evaluated according to the aforementioned criteria. Full texts were then analyzed in order to extract the following data: patients’ age; sex; previous RT on head and neck region; the presence of medical comorbidities; TNM stage; type of head and neck surgery (total laryngectomy or pharyngolaryngectomy) and the possible use of a free or pedicled flap for reinforcing the pharyngeal suture; time elapsed (expressed in days) from surgery to fistula presentation; time from fistula appearance to its closure; and other associated postoperative complications. 

### 2.3. Definition of the Outcome and Statistical Methods

The aim of this study was to quantify the success rate, defined as complete fistula closure, after the initiation of a non-surgical strategy and in addition to standard wound therapy. Standard descriptive statistics were used to summarize the extracted data and Microsoft^®^ Excel (Version 16.52, Redmond, WA, USA) was used to perform the calculations.

## 3. Results

**Figure 1:**
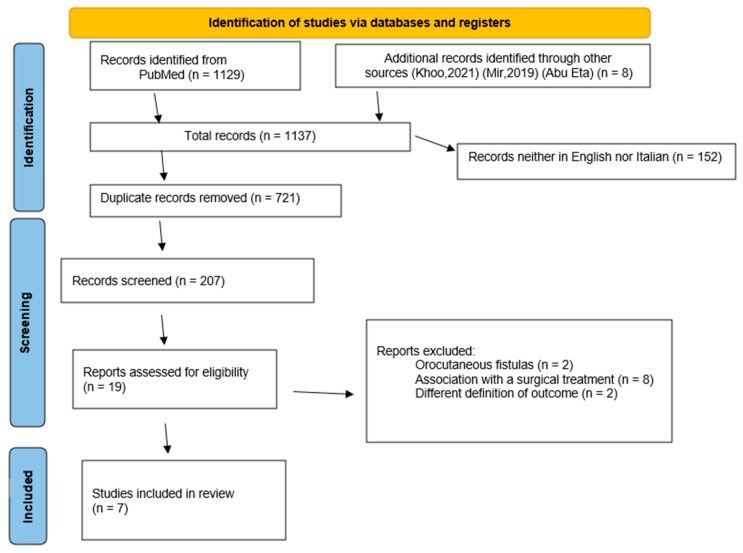
PRISMA flow diagram depicting the selection of the papers included in the present review.

After the application of selection criteria, a total of seven articles were included in the present review and the PRISMA flowchart is represented in Figure 1. Non-surgical strategies for PCF closure included botulinum toxin therapy (2 studies), HBOT (2 studies), and NPWT/VAC (3 studies) for a total of 27 patients. In all cases, these treatments were specifically added to the standard PCF care that included compressive dressings, systemic broad-spectrum antibiotics administration, and enteral artificial nutrition, usually by a nasogastric feeding tube.

In the whole cohort, there was only one female and the mean age was 63.3. Primary tumors’ stages ranged from T2N0 to T4N2, and 14/27 patients (51.9%) had previously received RT. PCF was diagnosed a mean of 9.6 days after surgery and it healed about 25.0 days after its appearance. Comorbidities were seldom reported and only a few (3) studies have specifically investigated them: diabetes mellitus (2), ischemic heart disease (2), hypertension (2), dyslipidemia (3), COPD (1), and atrial fibrillation (1).

The detailed outcomes for the use of HBOT, NPWT, and botulinum toxin therapy are presented in Table 1, Table 2 and Table 3, respectively. The overall success rate was 92.6% but no formal comparison with a control group was ever made. In most cases, conservative treatments were used primarily, but in 11% of cases, they were also used after failed surgical treatment (2 pectoralis major flap, 1 radial forearm free flap). The reason for choosing one therapeutic process rather than another was never explicitly motivated. Finally, we have stratified patients according to previous RT status: those who have never been irradiated (13) had a mean healing time of 14.5 days versus 35.4 days in the RT group. Success rates were therefore 100% versus 85.7%, but the statistical comparison could not be performed due to missing data.

## 4. Discussion

The optimal management of PCF begins preoperatively, with a proper assessment of the risk factors in each patient: poor nutritional status (measured by hypoalbuminemia), previous head and neck radiation therapy, or systemic chemotherapy can all increase the chance of PCF development [15]. These findings are probably due to the exacerbation of the obliterative endarteritis and fibrosis induced by the (chemo) radiation itself in local tissues [16]. While malnutrition should be aggressively corrected during the whole perioperative period [17], in the setting of a salvage laryngectomy/pharyngolaryngectomy the prophylactic use of a reinforcing flap has been shown to significantly decrease PCF risk [18]. In addition, while early oral feeding seems to increase PCF risk according to a recent meta-analysis [19], early oral hydration may actually reduce this probability (by a possible mechanical detersion of infected fluids and saliva on pharyngeal suture) [20].

Another point to remember is that preventing PCF development remains of the uttermost importance: a recent interesting experience has presented a “fistula-zero project” after total laryngectomy which is mainly based on a watertight horizontal pharyngeal closure, the reinforcing flap for post-RT patients, and the use of salivary bypass tubes [21]. Besides prevention, a key aspect is to make an early diagnosis of fistula. For instance, blue dye oral testing can help in intraoperative and early postoperative periods, with the advantage of being low in cost and easily administered [3]. Another early predictive test for PCF is represented by the presence of wound amylase in drains [22].

Once PCF appears, it is known that between 60% and 80% of cases will heal with “conservative treatments” [23]. Conventionally, a broad-spectrum antibiotic therapy is set up, along with compressive wound care, and enteral nutritional support through a nasogastric tube. Although they are not formally supported by the evidence, these treatments appear to be plausible and somehow even obvious [23,24]. Surgical repair remains the most effective and rapid way to close PCF, which, if left untreated, can favor deep neck infections and carotid blowout syndrome [5,25]. A recent multicenter study has even shown an independent association between PCF and the development of distant metastases, but the authors did not include in their model the delayed start of adjuvant treatments [26]. 

In the present review, we have shown that additional non-surgical strategies, such as HBOT, VAC, or botulinum toxin therapy, might yield an overall satisfactory PCF closure rate. Even though no side effects were reported with these strategies, we think that this result is heavily weakened by the lack of a control group, the fact that confounding factors, such as preoperative comorbidities or previous RT, were not evaluated, and the very low number of cases available. Furthermore, we strongly believe that these strategies should be reserved only in very particular cases and, whenever judged to be clinically necessary, prompt surgical closure should be performed [1,5].

HBOT is based on the favorable effects of repetitive periods of hyperoxia (and subsequent hypoxia) on the wound healing process and mainly thanks to the production of the vascular endothelial growth factor (VEGF) by macrophages [27]. The treatment is quite safe, even though some side effects were reported, including reversible myopia, barotrauma in the form of tympanic membrane perforation, tracheobronchial symptoms (from a simple cough to pneumothorax), and even seizures [28]. While the use of HBOT has become a cornerstone in other fields of head and neck surgery, such as in the case of mandibular osteonecrosis [29], we retrieved no satisfactory study for PCF treatment. In addition, HBOT for treating PCF raises some questions, such as the yet unassessed cost-benefit ratio, or the risk of promoting tumor progression, since oxygen was shown to promote cellular and vascular proliferation in wounds [30]. However, according to a review of the literature conducted some years ago, this latter risk remains more theoretical than clinically meaningful [31].

The efficacy of NPWT/VAC therapy for all kinds of head and neck wounds has been recently reviewed in an excellent paper [32]. One problem is the definition of the outcome. For example, one study using NPWT considered “success” the mere formation of granulation tissue in the PCF tract, without specifying any further [33]. Another issue arises from the difficulty to understand when NPWT can be applied non-invasively or when it needs to be placed in the operating room along with open surgical wound revision [34,35,36,37], or even by an endoscopic insertion [38]. Not all the patients of the considered articles were, therefore, included for this study. In two papers, for example, for some patients of the cohorts, an explicitly invasive surgical procedure was associated with VAC [10,11]. Furthermore, maintaining the necessary hermetic seal is notably difficult because of the proximity of the PCF to the tracheal stoma, whose secretions also complicate VAC adherence to the skin [11]. On the other hand, NPWT has got very few side effects other than pain/discomfort [39], or hemorrhage if vessels are not properly protected [40]. Regarding costs, it was shown they are comparable to those of conventional wound dressing, because of the need for fewer dressing changes and a shorter duration of hospitalization [41].

Botulinum toxin therapy represents one of the many pharmacological strategies to reduce salivary flow, which is a major culprit in PCF formation and persistence [6 bomeli]. This molecule has been extensively used in the treatment of sialorrhea for many neurological syndromes [42,43,44,45]. Botulinum toxin starts to reduce the salivary secretion from 72 h after the periparotid infiltration and it has a more noticeable effect after 5 to 7 days [46]; its action is reversible and it lasts about 2–4 months, and with minimal systemic side effects [47]. A study conducted some years ago demonstrated, by the use of 99 mTc pertechnetate scintigraphy, a reduction of up to 80% total salivary flow of secretion. However, it should be noted that some residual flow remains nonetheless important against oral cavity infections and xerostomia [48]. In addition, botulinum toxin use for PCF is currently off-label, and a comprehensive and written informed consent to the procedure should be obtained (as done by one [13] of the two aforementioned studies). Another potentially useful antisecretory drug would be constituted by scopolamine patches, which, however, are not free from anticholinergic side effects (e.g., blurred vision and urinary retention) [49,50]. However, in our literature search anticholinergic drugs were reported for the treatment of neurological chronic drooling [51,52], or for the prevention of sialocele or the treatment of salivary fistulas following parotid surgery [53,54], while no mention as a possible treatment for PCF was found. For the sake of completeness, we found that successful use of combination treatments, such as scopolamine + botulinum toxin for a post-parotidectomy fistula [55], or scopolamine + NPWT for treating an entero-cutaneous fistula after esophagectomy [56], was also presented in case reports.

The possible role of previous RT as a risk factor is well known since many years: the scientific evidence has been strengthened by several meta-analyses, even though some studies have shown contradictory results [1,15,57]. Furthermore, it has been shown that chemo-radiotherapy increases the risk of developing a PCF compared to radiotherapy alone [16]. It should be recalled that the group receiving salvage RT may receive more extensive procedures because of the higher recurrent T/N stage and the need for larger resection margins, and this latter aspect can increase, by itself, the risk of complications [58]. Since the publication of the aforementioned meta-analyses, it has become almost imperative to reinforce pharyngeal closure with a vascularized flap and many choices are available (pectoralis major flap, supraclavicular artery island flap, fasciocutaneous free flaps, mammary artery, perforator propeller flap, latissimus dorsi flaps, or facial artery-based cutaneous island flap) [59,60,61,62]. In a large multicenter study conducted by the American Academy of Otolaryngology-Head & Neck Surgery, vascularized tissue augmentation was shown to significantly reduce the overall fistula rate and fistula requiring reoperation, but, also, to possibly impair speech and swallowing outcomes [63].

Limitations of our study include the heterogeneity in terms of elapsed time from PCF diagnosis and the start of the therapy, in terms of indications, and in the definition of the outcome. For instance, Steffen et al. reported six patients with PCF and treated with botulinum toxin, speaking only of “improvement of the patients’ conditions”, and without mentioning the fistula closure [64]. Another issue arises from the lack of a control group: only the study by Neovius et al. had a reference group, but the authors did not make any statistical comparison between the two cohorts [8]. The diffuse underreporting (of comorbidities, of previous RT, etc.) of all of the available studies is the major reason for the low level of evidence of the present work. In addition, since it is based mostly on case series/reports, it is very probable that the success rate is high for the simple fact that only successful treatments have been reported (i.e., publication bias). Moreover, concomitant or subsequent multiple non-surgical treatments would not allow us to draw any conclusion on what, in the end, has helped in the healing process, such cases are, therefore, not useful for comparison against other surgical or single non-surgical treatments. Hopefully the present review will prompt future research in this field and we suggest that a basic set of clinical information must be reported with these treatments if we want to draw stronger clinical conclusions.

## 5. Conclusions

The outcomes of non-surgical strategies for expediting the closure of PCF seem apparently promising, but they are derived from only a few non-randomized and retrospective studies. The very low level of evidence available does not currently justify the use of these strategies in the current clinical management of PCF and further well-designed and exhaustive studies are needed in this field of head and neck surgery.

## Figures and Tables

**Table 1:** Overview of the clinical outcomes of patients treated for PCF using hyperbaric oxygen therapy (HBOT). Acronyms: NA, not available; M, male; F, female; PMF, pectoralis major myocutaneous flap; DM, diabetes mellitus; HTN, hypertension; IHD, ischemic heart disease; PAF, paroxysmal atrial fibrillation.

Reference (Year, Country)	Study Type (Period)	N° of Cases (Sex)	Mean Age	Tumor Site	cTNM/ pTNM	Type of Surgery	Previous RT (Number of Patients)	Previous Surgical Treatment for Fistula Healing	Comorbidities	Time for Fistula Presentation (Days after Surgery)	Mean Time for Fistula Healing (Days)	Success Rate (%)
Neovius et al. (1997, Sweden) [8]	Retrospective (1993–1995)	2 (2M/0F)	58.5	Laryngeal	T2-3 N0 M0	NA	2	NA	NA	NA	45	50%
R. Abu Eta et al. (2016, Israel) [9]	Retrospective (2008–2013)	8 (8M/0F)	63	Glottic-supraglottic	T3-4 N0-2b M0	8 total laryngectomy + 6 PMF	8	0	1 DMII 1 HNT 2 IHD 2 Dyslipidemia	12.75	41.5	87.5%

**Table 2:** Overview of the clinical outcomes of patients treated for PCF using negative pressure wound therapy (NPWT). Acronyms: NA, not available; M, male; F, female; PMF, pectoralis major myocutaneous flap; RFFF, radial forearm free flap; DM, diabetes mellitus; HTN, hypertension.

Reference (Year, Country)	Study Type (Period)	N° of Cases (Sex)	Mean Age	Tumor Site	cTNM/ pTNM	Type of Surgery	Previous RT (Number of Patients)	Previous Surgical Treatments for Fistula Healing	Comorbidities	Time for Fistula Presentation (Days after Surgery)	Mean Time for Fistula Healing (Days)	Success Rate (%)
Andrews et al. (2008, USA) [10]	Case series (NA)	1 (1M/0F)	75	larynx	T3-N0-M1	Salvage + radial forearm reconstruction	1	0	NA	NA (dehiscence soon after the surgery)	28	100%
Loaec et al. (France, 2014) [11]	Case series (2011–2013)	5 (5M/0F)	67.4	Larynx-oropharynx	T3-4 N0-2 M0	1 total laryngectomy; 1 total circular pharyngolaryngectomy + RFFF; 2 partial laryngectomies, 1 transmandibular oropharyngectomy	0	1 reoperation for Hematoma + debridement (NPWT for the recurrence of fistula) 1 RFFF	1 COPD	7.6	17.8	100%
Teixeira et al. (2017, Portugal) [12]	Case series (NA)	2 (1M, 1F)	64	Pyriform sinus	pT3N0M0	2 total laryngectomies (+1 RFFF)	1	1 PMF	1DMII+, HNT obesity, splenectomy nephrectomy in the context of polyarteritis nodosa	NA	22	100%

**Table 3:** Overview of the clinical outcomes of patients treated for PCF using botulinum toxin therapy. Acronyms: NA, not available; M, male; F, female; PMF, pectoralis major myocutaneous flap.

Reference (Year, Country)	Study Type (Period)	N° of Cases (Sex)	Mean Age	Tumor Site	cTNM/ pTNM	Type of Surgery	Previous RT (Number of Patients)	Surgical Treatment for Fistula Healing	Comorbidities	Time for Fistula Presentation (Days after Surgery)	Mean Time for Fistula Healing (Days)	Success Rate (%)
Marchese et al. (2008, Italy) [13]	Case series (2004–2006)	6 (6M/0F)	62.5	Larynx-hypopharynx	NA	NA	1	0	NA	7	6.7	100%
Guntinas-Lichius et al. (2002, Germany) [14]	Case series (NA)	3 (3M/0F)	58	Larynx-oropharynx	rpT4 N2a-b M0	2 Laryngectomy + neck dissection; 1 Median mandibulotomy, tumor resection, radial forearm flap, neck dissection	1	1 PMF (failed)	NA	9.6	23.3	100%

## Data Availability

The data presented in this study are available upon reasonable request from the corresponding author.
